# Adoption and Performance of Complementary Clinical Information Technologies: Analysis of a Survey of General Practitioners

**DOI:** 10.2196/16300

**Published:** 2020-07-23

**Authors:** Placide Poba-Nzaou, Sylvestre Uwizeyemungu, Xuecheng Liu

**Affiliations:** 1 Department of Organization and Human Resources University of Quebec in Montreal Montreal, QC Canada; 2 Accounting Department University of Quebec in Trois-Rivières Trois-Rivières, QC Canada; 3 Statistics Department 12M Statistical Consulting Services Montreal, QC Canada

**Keywords:** electronic health record, personal health record, health information exchange, telehealth, general practitioners, quality of care, efficiency, organizational productivity

## Abstract

**Background:**

The benefits from the combination of 4 clinical information systems (CISs)—electronic health records (EHRs), health information exchange (HIE), personal health records (PHRs), and telehealth—in primary care depend on the configuration of their functional capabilities available to clinicians. However, our empirical knowledge of these configurations and their associated performance implications is very limited because they have mostly been studied in isolation.

**Objective:**

This study aims to pursue 3 objectives: (1) characterize general practitioners (GPs) by uncovering the typical profiles of combinations of 4 major CIS capabilities, (2) identify physician and practice characteristics that predict cluster membership, and (3) assess the variation in the levels of performance associated with each configuration.

**Methods:**

We used data from a survey of GPs conducted throughout the European Union (N=5793). First, 4 factors, that is, EHRs, HIE, PHRs, and Telehealth, were created. Second, a cluster analysis helps uncover clusters of GPs based on the 4 factors. Third, we compared the clusters according to five performance outcomes using an analysis of variance (ANOVA) and a Tamhane T2 post hoc test. Fourth, univariate and multivariate multinomial logistic regressions were used to identify predictors of the clusters. Finally, with a multivariate multinomial logistic regression, among the clusters, we compared performance in terms of the number of patients treated (3 levels) over the last 2 years.

**Results:**

We unveiled 3 clusters of GPs with different levels of CIS capability profiles: *strong* (1956/5793, 37.36%), *medium* (2764/5793, 47.71%), and *weak* (524/5793, 9.04%). The logistic regression analysis indicates that physicians (younger, female, and less experienced) and practice (solo) characteristics are significantly associated with a weak profile. The ANOVAs revealed a strong cluster associated with significantly high practice performance outcomes in terms of the quality of care, efficiency, productivity, and improvement of working processes, and two noncomprehensive medium and weak profiles associated with medium (equifinal) practice performance outcomes. The logistic regression analysis also revealed that physicians in the weak profile are associated with a decrease in the number of patients treated over the last 2 years.

**Conclusions:**

Different CIS capability profiles may lead to similar equifinal performance outcomes. This underlines the importance of looking beyond the adoption of 1 CIS capability versus a cluster of capabilities when studying CISs. GPs in the strong cluster exhibit a comprehensive CIS capability profile and outperform the other two clusters with noncomprehensive profiles, leading to significantly high performance in terms of the quality of care provided to patients, efficiency of the practice, productivity of the practice, and improvement of working processes. Our findings indicate that medical practices should develop high capabilities in all 4 CISs if they have to maximize their performance outcomes because efforts to develop high capabilities selectively may only be in vain.

## Introduction

### Background

Over the past several years, a consensus has emerged on the recognition of the potential of clinical information systems (CISs) to improve the health care delivered to patients and save lives [1,2].

Electronic health records (EHRs) are at the heart of the reform of health systems in many developed countries [3] as well as middle-income countries such as Brazil [4] or India [5]. An EHR can be defined as “an electronic record of health-related information on an individual that conforms to nationally recognized interoperability standards, and that can be created, managed, and consulted by authorized clinicians and staff across more than one healthcare organization” [6]. EHRs can “help address the dual problems of high costs and poor quality in health” [7]. For instance, EHRs can assist clinicians to improve the care provided to patients by promoting adherence to guidelines [8], improving medical practice management, saving time, and facilitating condition-specific queries, to name a few [9]. In the specific context of primary health care organizations or general practices, EHRs offer “a unique opportunity to collect a wide range of ecologically valid patient data to support understanding of disease burden and health trajectories over the life-course” [10].

Due to their potential benefits, previous decades have witnessed rapid growth in the adoption of EHRs in health care settings. However, despite considerable investments by governments, the adoption of EHRs in some primary care organizations has been slow, especially in small practices [11], and disparities have been observed in terms of benefits associated with CISs in primary care practices [12]. In addition, other researchers have underscored that the potential benefits of EHRs are limited when health information stored in the system is shared only within the host institution, which means that greater benefits will be realized if only health information is shared beyond the host institution [13,14] and the technology used to support such sharing is health information exchange (HIE). For this reason, the *Meaningful Use* program in the United States includes incentives for health care providers to participate in HIE [15]. Empirical evidence suggests that the exchange and sharing of patient data can decrease mortality, systemic costs, and utilization costs in the emergency department [13,16,17].

In addition to the abovementioned CISs (EHR and HIE), the personal health record (PHR) has recently been gaining attention because of its potential to support the transformation of health systems to a more patient-centered model of care [18] as well as its key role in patient engagement [19,20]. Indeed, patient engagement is recognized as a critical factor for improving the quality of care [19] and patient safety [19,21,22]. It is not surprising, then, that patient engagement measures and the sharing of health information between providers and patients are also key parts of the *Meaningful Use* program in the United States [18,23]. Although the role of patients in the health care process is being increasingly recognized, Krist and Woolf [24] have pointed out that much of the energy of the health information movement has been devoted to the use of health information by clinicians, even though patients’ use of these technologies carries equal promise. One of the most effective ways to share electronic health data with patients is via PHRs.

Similarly, telehealth has been gaining attention because of its potential to reduce barriers to access health care and to save time and reduce costs for remote patients [25,26]. In addition, telehealth can “be clinically supportive and educative by facilitating contact with peers” and, in turn, education can enhance the quality of care provided to patients [27,28].

In conclusion, EHR, HIE, PHR, and telehealth can be considered the most important components of a modern and desirable CIS for both hospital and primary care practices. However, in previous research, these 4 CISs have been studied in isolation with little or no attention to their combination and associated implications for performance outcomes. A search in Medical Literature Analysis and Retrieval System Online (MEDLINE); March 2020) using the 4 terms, “*electronic health records*,” “*health information exchange*,” “*personal health record,*” and “*tele-health*,” in all fields yielded no hits. The same search performed in *Article title*, *Abstract*, *Keywords* in Scopus (March 2020) yielded no hits. We conclude that there is some evidence from which one can infer that the body of knowledge in the way capabilities associated with the 4 CISs empirically coexist to form *configurations* or *profiles,* and the associated implication for performance outcomes is very limited. Of note is the fact that most studies have investigated the 4 CISs in isolation, including their implications for performance outcomes. For instance, several current reviews have found ambivalent or no significant relationship between EHRs and performance outcomes in primary care settings [29,30]. For their part, Black et al [29] concluded that “there is a large gap between the postulated and empirically demonstrated benefits of eHealth technologies [such as EHR, HIE, PHR, and telehealth].”

This paper takes a configurational perspective. In a broad sense, configuration is defined as “any multidimensional constellation of conceptually distinct characteristics that commonly occur together” [31].

Following Miller [32]; Fiss, Marx, and Cambré [33]; and Delery and Doty [34], we argued that studying the configurations of CIS adoption by primary care practices will not only allow us to take a holistic view of the adoption of CIS by these organizations as it is these patterns or profiles of 4 CISs rather than single isolated CISs that are related to performance, but it will also help to reveal insights that would have been otherwise difficult to obtain. In addition, configuration approaches help to investigate *equifinality—*defined as a concept in which different primary care practice profiles with different configurations of CIS adoption arrive at the same level of the outcome measure in terms of practice performance outcomes [35].

### Objectives

This study has 4 primary goals. The first is to identify predictors of the adoption of clinical systems by general practitioners (GPs) in Europe. The second is to identify and characterize GPs by uncovering typical profiles or patterns of the combination of 4 major CIS capabilities: EHRs, HIEs, PHRs, and telehealth. Consequently, this second objective is inductive in nature, empirically based, and taxonomic, dedicated to classification and subdivision [36,37]. The third objective is to identify physician and practice characteristics that predict cluster or profile membership. Finally, given that the ultimate objective of investing in CISs is to improve the quality of care provided to patients while decreasing cost, the fourth objective is to assess the variation in levels of performance outcomes associated with practice in each configuration or profile.

## Methods

### Data Source and Sample

We used a data set provided by the European Commission (EC) from the 2018 survey of European GPs. The objective of this study was to understand and measure the actual adoption and use of information and communication technology (ICT) and electronic health (eHealth) applications by general practitioners (GPs) in the 27 countries of the European Union (EU27) as well as changes in uptake over time. The 2018 survey of European GPs was a follow-up study of the 2013 survey, which included the EU27 plus 4 other countries (Croatia, Iceland, Norway, and Turkey).

Given that this 2018 survey of European GPs was a follow-up of the 2013 survey, eHealth is broadly defined, as in the previous study, as “the use of Information and Communication Technologies (ICT) across the whole range of health care functions” [38]. The 2018 survey used the same methodological approach and questionnaire as the previous study. Data collection was based on a survey in the EU27 using mixed methodology (web-based; web CATI, where CATI stands for computer-assisted telephone interviews; and face-to-face) and was conducted between January and June 2018 [38,39]. The data collection process was endorsed by the European Union of General Practitioners. A detailed description of the methodological approach is provided only in the 2013 survey report. The EC team took several measures to ensure the representativeness of the sample. The details are provided in the Technical Compendium of 121 pages published by the EC and available to the public [38]. The identification of the universe of the survey was based on the definition from the World Organization of Family Doctors Europe that characterizes GPs in European countries as “specialist physicians trained in the specialty of primary care who ‘exercise their professional role by promoting health, preventing disease and providing cure, care, palliation and promoting patient empowerment and self-management’.” The universe was composed of 425,622 GPs [39].

The questionnaire was composed of 3 parts: (1) GPs’ sociodemographics, organizational settings, practice location, description of tasks, and workload; (2) ICT availability and use within a GP practice that is divided into 4 categories: EHRs, HIE, telehealth, and PHRs; and (3) attitudinal questions as well as questions related to motivations, perceived barriers, and impacts of ICT.

Following the previous 2013 survey approach, a final sample of 5793 GPs was randomly selected over the analyzed EU27, with an overall sampling error of plus or minus 1.30% [39].

Given that the 4 objectives of this study are related to CIS adoption and associated with the implications for performance outcomes, out of the initial sample of 5793 GPs, only the 5244 who had an EHR system and stored patient data electronically were considered for a subsequent analysis. Due to the presence of missing values in 3 variables (HIE, PHR, and telehealth), we applied a multiple imputation strategy. Among the 5244 subjects, 5022 (95.77%) subjects had complete data, 100 (1.91%) subjects had missing values on telehealth only, 72 (1.37%) subjects had missing values on PHR only, and the remaining 50 (<1%) subjects had other missing patterns.

We imputed the missing values using a multiple imputation procedure. On the one hand, multiple imputation methods perform better than single imputation ones. We selected the multiple imputation method of fully conditional specification with the Proc MI procedure in SAS software version 9.4 (SAS Institute) because of its flexibility in allowing us to define the multivariate model by a series of conditional models, one for each incomplete variable [40]. The 4 variables related to each CIS were chosen for the imputation model: EHR, HIE, telehealth, and PHR. For each of the 4 variables, we then chose regression because the regression model allows the specification of a minimum and a maximum value for imputed values on a variable-by-variable basis [41].

On the other hand, it is impossible to use multiple imputed data sets for cluster analysis as it produces different results of clusters, and it seems that there are no methods to combine these results. Therefore, we used the multiple imputation method as discussed but only chose *one* as the number of imputations. The final 4 variables used in the cluster analyses were the ones after imputations and then standardization: xc_EHR_imp_std, xc_HIE_imp_std, xc_TeH_imp_std, xc_PHR_imp_std.

Although the final 4 variables used for the cluster analysis were the ones with an imputation, we performed the same cluster analysis with the same variables without the imputation of missing values.

As stated earlier, the 549 GPs who did not store patients’ data electronically and those who did not have an EHR were excluded from the analysis. The final sample in our analysis was 5793 – 549 = 5244 subjects. Table 1 compares the characteristics of the 549 and 5244 subsamples. Concerning the total sample (N=5244) composition in terms of countries, 8 countries represent a little less than 50% (49.77%) of the sample, each country accounting for about 5.54% and 7.15% of the sample, respectively. These countries include France, Italy, Poland, Portugal, Romania, Spain, the United Kingdom, and the Netherlands.

**Table 1:** Characteristics of the respondents and their practices.

Variables, characteristics (ie, levels for categorical variables)		Nonsampled (n=549)	Sampled (n=5244)	Chi-square (*df*)	*t* test (*df*)	*P* value
**Gender, n (%)**						
	Male	247 (45.0)	2652 (50.57)	62 (1)	N/A^a^	.01
	Female	302 (55.0)	2592 (49.43)	62 (1)	N/A	.01
Age (years), mean (SD)		53.23 (10.35)	51.86 (10.82)	N/A	2.8 (5791)	.005
Years spent in general practice, mean (SD)		21.07 (11.13)	20.83 (11.22)	N/A	0.5 (5791)	.64
**Professional status, n (%)**						
	Working in a health center	159 (29.0)	1547 (30.02)	103.0 (3)	N/A	<.001
	Self-employed GPs^b^ working alone	272 (49.5)	2013 (38.39)	103.0 (3)	N/A	<.001
	Self-employed GPs working in a group practice	37 (7)	1226 (23.38)	103.0 (3)	N/A	<.001
	Other	81 (15)	431 (8.2)	103.0 (3)	N/A	<.001
**Size of the practice (number of physicians), n (%)**						
	Solo (1)	320 (58.3)	2155 (41.09)	67.4 (3)	N/A	<.001
	Small (2-4)	58 (11)	920 (17.5)	67.4 (3)	N/A	<.001
	Medium (5-9)	60 (11)	995 (19.0)	67.4 (3)	N/A	<.001
	Large (10 or more)	111 (20.2)	1174 (22.39)	67.4 (3)	N/A	<.001
**Location of workplace, n (%)**						
	Large city	204 (37.2)	1944 (37.07)	1.0 (2)	N/A	.62
	Medium- to small-sized city	156 (28.4)	1402 (26.74)	1.0 (2)	N/A	.62
	Rural town	189 (34.4)	1898 (36.19)	1.0 (2)	N/A	.62

^a^N/A: not applicable.

^b^GPs: general practitioners.

### Selection of Clustering Variables and Measurements

As recommended by Aldenderfer and Blashfield [42] and Hair et al [37], the selection of clustering variables is theory driven. The selection of variables was based on the fact that they have a common theoretical foundation—they relate to the functional characteristics or capabilities of 1 of the 4 CISs (EHR, HIE, PHR, and telehealth). Indeed, according to the diffusion of innovation theory (DOI) [43], the characteristics of innovation, such as the functional capabilities of CISs are important factors in explaining its adoption.

All clustering variables were dichotomous (1: available and 0: not available). In total, 44 dichotomous variables were selected based on their theoretical relationship; that is, they relate to the functional characteristics of 1 of the 4 CISs (EHR, HIE, PHR, or telehealth). We also used 5 measures of performance. Four measures (quality of care provided to patients, efficiency of the practice, productivity of the practice, and improvement of working processes) were based on a 4-point Likert-type scale ranging from 0 (“strongly disagree”) to 3 (“strongly agree”), and 1 measure (number of patients over the last 2 years) of a categorical type with 3 categories (1: decrease, 2: remain the same, 3: increase)

A standardized average frequency or scaled frequency was computed for each group of variables, including CIS capabilities and performance, resulting in 8 scales. As presented in Table 2, in 7 out of 8 scales, the measurement of reliability is greater than 0.7 [44]. Although the reliability of telehealth was less than 0.7, we decided to keep this factor because Cronbach’s alpha is sensitive to the number of items in the scale and generally tends to underestimate the internal consistency reliability” [45] and “researchers can rely on less stringent reliability measurements for scales consisting of a few items” [46]. We then used standardized average frequencies as the clustering variables.

**Table 2:** Reliability of the scales.

Factors		Number of items		Cronbach alpha	
**Clinical system capabilities**					
	Electronic health record		19		.87
	Health information exchange		15		.87
	Telehealth		4		.59
	Personal health record		6		.80
**Performance measurement**					
	Quality of care provided to patients		6		.92
	Efficiency of the practice		5		.89
	Productivity of the practice		5		.83
	Improvement of personal working practice		4		.80

The statistical analysis was performed in 3 parts: (1) cluster analysis, (2) ANOVAs With Tamhane T2 Post Hoc Tests, and (3) regression analysis.

### Statistical Analysis 1: Cluster Analysis

As indicated earlier, we adopted a configurational approach that is taxonomic, based on cluster analysis [31,47]. General practices were classified to reveal patterns based on the available CIS capabilities related to EHR, HIE, PHR, and telehealth. Thereafter, we explored the influence of general practitioner and practice characteristics on the availability of the 4 components of CISs included in this study (EHR, HIE, PHR, and telehealth).

Broadly speaking, cluster analysis is a multivariate statistical technique that classifies items or objects (individuals, firms, or behaviors) from a given population into subgroups or clusters so that items that are classified in the same cluster are more similar to one another than they are to items in another cluster [37,48]. In doing so, the homogeneity of objects within each group is maximized, whereas the heterogeneity between the groups is maximized. Cluster analysis is a completely empirical method of classification that is inductive by nature [49]. Following previous research recommendations [48-50], our procedure was based on 2 steps that allowed the determination of the natural number of clusters in the data set, as it has been found to be more effective than other approaches in the ability to recover clusters. First, a hierarchical algorithm was employed to identify and define natural clusters and associated centroids. These centroids were then used as initial seeds in a nonhierarchical algorithm. Later, a discriminant function analysis was carried out [49] to validate the cluster solution. Several studies have used this technique to validate the results of cluster analysis [51,52].

It is important to remember that contrary to other statistical techniques such as regression analysis, which necessitate the satisfaction of the linearity assumption and have established rules for sample size calculation [53], no such assumptions are necessary for cluster analysis [54]. Given that this is a data mining technique, cluster analysis does not have *hard* sample size rules and does not need to satisfy parametric or even nonparametric statistical test assumptions [55]. Nonetheless, Hair et al [56] underscored that “the sample size must be large enough to provide sufficient representation of small groups within the population and represent the underlying structure.” For their part, Formann [57,58] as well as Dolnicar [59] contend that there is a consensus that the minimal sample size for cluster analysis is 2^k^ observations (k=number of variables) to achieve sufficient power and confidence in the statistical analysis. In the same vein, Lowry et al [55], including Dolnicar [59], indicate that from a conservative perspective, the minimal sample must be no less than 5×2^k^. In this study, k=4, which requires a minimum sample size of 80 to meet this criterion, which is obviously less than the sample size of this study (N=5244).

#### Determination of the Number of Clusters

The optimum number of clusters was determined by inspecting the dendrogram generated in the combination of the Ward minimum variance clustering algorithm and the squared Euclidean distance. This examination revealed that a 3-cluster solution would be optimal. To ascertain the reliability of the solution [48], the procedure was performed with different subsamples that were randomly selected (30%, 40%, and 60%). On the grounds of the preceding analysis, the 3-cluster solution was retained. It was the most meaningful and the one that best captured the patterns of the adoption of functional capabilities of EHR, HIE, PHR, and Telehealth among European GPs. The uncovered clusters formed significantly well-separated groups that had strong EHR, HIE, PHR, and telehealth functional capability adoption meanings. The 3 different patterns identified reveal how CIS capability priorities set at macrolevels (countries or EC), actually and empirically manifest at primary care levels.

It is worth recalling that cluster analysis was performed with and without the imputation of missing values. Of note is the fact that the results were similar with and without the imputation of missing values. As cluster analyses necessitate all variables to have nonmissing values, 740 observations were not classified in the analysis without the imputation of missing values. The two 3-cluster solutions were then compared to determine the degree of agreement among members of each cluster using Cohen kappa coefficient. The results reveal an almost perfect agreement between the two 3-cluster solutions with a kappa of 0.99 (kappa in the range of 0.80-1.00) [60].

#### Validation of the Cluster Solution and Multiple Discriminant Analysis

A multiple discriminant function analysis was performed following a cross-validation approach [37] to validate the 3-cluster solution and determine how the clusters differed on the 4 clustering variables portraying GPs based on EHR, HIE, PHR, and Telehealth functional capabilities [49]. Our discriminant analysis produced 2 functions with significant Wilks lambdas (*P*<.001). In addition, the hit ratios for the analysis and holdout samples were 95.90% and 94.70%, respectively. Two standard criteria were used to evaluate the accuracy of the hit ratios: the maximum chance criterion (C_max_) and the proportional chance criterion (C_pro_) [45]. The last authors [45] underscored C_max_ as the most conservative standard in that it will produce the highest standard of comparison. In this case, because the 3 clusters have different sizes, the C_max_ is equal to 52.70%. Hair et al [45] suggested that the classification accuracy should be at least one-fourth greater than the accuracy of the classification achieved by chance (1.25×C_pro_): C_pro_=43% and 1.25×C_pro_=53.75%. Overall, in a conservative view, both hit ratios are greater than maximum 1.25 (C_pro_ and C_max_). In conclusion, the null hypothesis (that the percentage correctly classified was not significantly different from what would be classified by chance alone) was rejected. Finally, from the distribution of the GPs along with the clusters’ centroids in the plane of the 2 computed discriminant functions (ie, the scatter plot of GPs in the space of the 2 discriminant functions with the clusters’ centroids), we were able to observe a complete separation of each one of the centroids from another along the 2 discriminant axes [61]. The evidence suggests that the discriminant functions performed very well in separating the 3 groups.

Although our empirically derived taxonomy appears to be meaningful, its quality is discussed in light of Rich [62] framework on requirements for a valid taxonomy: breadth, meaning, depth, theory, quantitative measurement, completeness, logic, and recognizability. Following the call by Dayer et al [63] to adapt the framework when applied to taxonomies created through cluster analysis, the assessment is based on 5 of the 7 initial criteria.

##### Breadth

The selection of clustering variables from which GPs were grouped was theory driven [37,42]. As stated earlier, the 18 clustering variables were selected based on their having a common theoretical foundation, that is to say, they pertain to the functional characteristics of 1 of the 4 CISs (EHR, HIE, PHR, or Telehealth), and previous studies have confirmed the influence of these clustering variables on the adoption of technologies [43,64,65].

##### Meaning

The resulting taxonomy is built on the broad foundations of the DOI theory [43], which acknowledges the complexity of the adoption of innovation and suggests that GPs should not be considered a homogeneous group when investigating the adoption of CIS capabilities. This theory supports the need for classification of the GPs adopting CIS based on the capabilities they actually adopted. The resulting taxonomy highlights different clear priorities and routes to the adoption of the 4 major CIS capabilities.

##### Theory

The anchoring of the development of our taxonomy in the theory of DOI [43] provides a robust theoretical foundation that serves as a qualitative basis for GP grouping justification as well as variable selection and helps to describe and understand the adoption of the 4 major CISs (EHR, HIE, PHR, and Telehealth) at the medical practice level.

##### Quantitative Measurement

GPs were assigned to specific clusters or groups resulting from an inductive process based on empirical, multivariate data analysis as well as the application of ANOVA and post hoc analysis to enhance the validity of the results.

##### Recognizability

By deriving the taxonomy from the actual capabilities of the CIS circumscribed by each artifact (EHR, HIE, PHR, and telehealth technologies), we can claim that our taxonomy reflects the real world for both practitioners as well as theorists and depicts the actual landscape of EHR, HIE, PHR, and Telehealth adoption by GPs within the EU.

In a subsequent step, the 3 profiles (clusters) were compared according to 4 performance outcomes (quality of care provided to patients, efficiency of the practice, productivity of the practice, improvement of working processes) using an ANOVA and a Tamhane T2 post hoc test.

### Statistical Analysis 2: ANOVAs With Tamhane T2 Post Hoc Tests

We used ANOVAs with Tamhane T2 post hoc tests to compare the 4 performance outcomes that were rated with a Likert scale: quality of care provided to patients, efficiency of the practice, productivity of the practice, and improvement of personal working practice.

### Statistical Analysis 3: Regression Analysis

This analysis was performed in 4 steps. First, we used a regression model to identify the predictors of CIS adoption.

Second, for each of the following 6 characteristic variables, that is, 4 physician characteristics (gender, age, professional status, and years spent in general practice) and 2 practice characteristics (workplace location and practice size), a univariate multinomial logistic regression was conducted to analyze the effect of the characteristic variable on cluster membership.

Third, the multivariate multinomial logistic regression model was conducted with the 6 characteristic variables as independent variables and the 3-cluster solution as the outcome variable. This was used to analyze the effect of each characteristic variable on cluster membership, as shown in the first step, but controlling for the other 5 characteristic variables.

Fourth, the multiple multinomial logistic regression was performed to see if the cluster membership predicted the level of performance in terms of “the number of patients over the past two (2) years” when controlling for the 6 characteristic variables.

## Results

### Characteristics of Adopters Versus Nonadopters of Electronic Storage of Patient Data

The logistic regression model indicates that compared with GPs who store patient data electronically, those who do not tend to be older in age, self-employed, working alone, with fewer years spent in general practice (Multimedia Appendix 1). Of note is the fact that the odds ratios (ORs) for age and *years spent in general practice* are close to 1.

### Empirical CIS Profiles of GPs

As shown in Table 3, the ANOVA *F* test is highly statistically significant for all 4 factors or groups of functional capabilities. In addition, the Tamhane T2 post hoc multiple pairwise comparison test revealed significant differences between the means of all 4 factors or groups of functional capabilities across the 3 clusters [66]. It is important to recall that, contrary to other tests such as the Tukey Honestly Significant Difference Test, the Tamhane T2 post hoc test does not assume equal variances.

**Table 3:** Capabilities profile and analysis of variance of clinical information systems.

Variables		Cluster			ANOVA^a^	
		1	2	3	*F* test (*df*)	*P* value
Number of participants (n=5244), n (%)		1956 (37.30)	2764 (52.71)	524 (10.0)	N/A^b^	N/A
**Clinical system capabilities^c^**						
	Electronic health record	*H:* 0.39^d^	*M:* 0.19^e^	*L:* −2.47^f^	5675.8 (2)	<.001
	Health information exchange	*H:* 0.88^d^	*M:* −0.42^e^	*L:* −1.05^f^	2517.1 (2)	<.001
	Telehealth	*H:* 0.62^d^	*M:* −0.37^e^	*M:* −0.33^f^	764.7 (2)	<.001
	Personal health record	*H:* 0.93^d^	*M:* −0.55^e^	*M:* −0.56^f^	2727.4 (2)	<.001

^a^ANOVA: analysis of variance.

^b^N/A: not applicable.

^c,d,e,f^Within rows, different superscripts indicate significant (*P*<.05) pairwise differences between means on Tamhane T2 post hoc test. H=high; M=medium; L=low.

#### Cluster I

The strong profile (n=1956) is the second largest of the 3 clusters and accounts for approximately 37% of the sample. Statistically, this cluster scored *high* in the pairwise difference between the means of all 3 groups for all 4 CIS capabilities. GPs within this cluster have a strong CIS capability profile and pay a great deal of attention to all 4 CISs, namely EHR, HIE, PHR, and telehealth. Thus, cluster I is named the *strong* CIS capabilities profile.

#### Cluster II

The moderate profile (n=2764) is the largest of the 3 clusters and accounts for approximately 53% of the sample. This cluster scored *medium* in the pairwise difference between the means of all 4 CIS capabilities. GPs within this cluster have an equally moderate emphasis on all 4 CIS capabilities. Thus, cluster II is named the *moderate* CIS capabilities profile.

#### Cluster III

The weak profile (n=524) is the smallest of the 3 profiles and accounts for approximately 10% of the sample. This cluster scored *low* in the pairwise difference between the means for 2 of the 4 CIS capabilities (EHR and HIE), whereas scoring *medium* for Telehealth and PHR capabilities. GPs within this cluster exhibit a focus on Telehealth and PHR capabilities, albeit with moderate strength. Thus, cluster III is named the *weak* CIS capabilities profile.

### Predictors of CIS Profile Membership

Univariate and multivariate logistic regression analyses were performed (Multimedia Appendix 2) with CIS profiles as the outcome and 6 independent variables of physician and practice characteristics: gender, age, physician professional status, workplace location, years spent in general practice, and practice size). Of note is the fact that the outcome variable has 3 CIS profiles, and the odds in the Multimedia Appendix are to the *strong* profile as the reference.

#### Physician Gender

The multivariate model indicated that female GPs were more likely than their male counterparts to be in the *weak* (OR 1.52, 95% CI 1.24-1.87) or *moderate* profiles (OR 1.18, 95% CI 1.04-1.33) when controlling for other GPs’ individual (age, physician status, and years spent in general practice) and practice (workplace location and practice size) characteristics.

#### Physician Professional Status

The multivariate logistic model indicated that the GPs working in a health center were less likely than the self-employed GPs working alone to be in the *weak* profile (OR 0.46, 95% CI 0.24-0.87) or *moderate* profile (OR 0.65, 95% CI 0.42-0.99) when controlling for other physicians and practice characteristics.

Similarly, self-employed GPs working in a group practice were found to be less likely than the self-employed GPs working alone to be in the *weak* profile (OR 0.44, 95% CI 0.22-0.87). However, there was no association between the 2 professional status and membership in the *moderate* profile. The same result is obtained when comparing GPs working neither with a health center nor as self-employed GPs in a group practice with the self-employed GPs working alone in relation to membership with any of the nonstrong profiles.

#### Physician Age

The multivariate model indicates no association between physician age and membership in the *weak* profile. However, the multivariate model indicates that older GPs are less likely to be in the *moderate* profile, with the OR decreasing to 0.97 (95% CI 0.96-0.98) when controlling for other characteristics.

#### Years Physicians Spent in General Practice

The multivariate model indicates that senior GPs are less likely to be in the *weak* profile, with the OR decreasing to 0.97 (95% CI 0.96-0.99) when controlling for other characteristics.

The multivariate model also indicates that GPs with more years of practice are more likely to be in the *moderate* profile, with the OR increasing to 1.02 (95% CI 1.00-1.03) for each additional year spent in practice when controlling for other characteristics.

#### Workplace Location

The multivariate model indicates that GPs within a practice located in a medium- to small-sized city or in a rural town are less likely than those located in a large city to be in the *weak* profile, with OR 0.741 (95% CI 0.58-0.95) and OR 0.60 (95% CI 0.48-0.77), respectively. No significant relationship was found between GPs more or less likely to be in the *moderate* profile when comparing workplaces either between medium- to small-sized cities and large cities or between rural towns and large cities.

#### Practice Size

First, no association was found for membership to the *moderate* profile when comparing small practice groups with those working in a solo practice.

Second, between medium and solo, GPs working in medium-sized practice groups are less likely to be in the *weak* profile and the *moderate* profile than those in a solo practice, with OR 0.34 (95% CI 0.18-0.66) and OR 0.46 (95% CI 0.30-0.71), respectively.

Finally, between large and solo, GPs working in large practice groups are also less likely to be in the *weak* profile and the *moderate* profile than those in solo practice, with OR 0.37 (95% CI 0.19-0.69) and OR 0.37 (95% CI 0.24-0.56), respectively.

### Comparison of CIS Profiles According to Practice Performance

As stated earlier, we used 5 measures of performance, including 4 based on a 4-point Likert-type scale (quality of care provided to patients, efficiency of the practice, productivity of the practice, improvement of working processes) and 1 variable of categorical type that is composed of 3 levels (1: decrease, 2: remain the same, 3: increase) related to the number of patients treated over the last 2 years.

The results in Table 4 indicates that the *strong* profile outperforms the other 2 in terms of the quality of care provided to patients, efficiency of the practice, productivity of the practice, and improvement of working processes. Surprisingly, however, the *moderate* and *weak* profiles do not differ from one another in terms of the quality of care provided to patients, efficiency of the practice, and productivity of practice, whereas the *moderate* profile outperforms the *weak* profile concerning improvement of working processes.

**Table 4:** Clinical information systems profiles and practice performance.

Variables		Cluster			ANOVA^a^		
		1	2	3		*F* test (*df*)	*P* value
Number of participants (n=5244), n (%)		1956 (37.30)	2764 (52.71)	524 (10.0)		N/A^b^	N/A
**Performance^c^**							
	Quality of care provided to patients	*H*: 0.18^d^	*M*: −0.09^e^	*M*: −0.20^e^		53.7 (2)	<.001
	Efficiency of the practice	*H*: 0.14^d^	*M*: −0.07^e^	*M*: −0.15^e^		33.3 (2)	<.001
	Productivity of the practice	*H*: 0.16^d^	*M*: −0.09^e^	*M*: −0.13^e^		41.0 (2)	<.001
	Working processes improvement	*H*: 0.18^d^	*M*: −0.06^e^	*L*: −0.37^f^		73.1 (2)	<.001

^a^ANOVA: analysis of variance.

^b^ N/A: not applicable.

^c,d,e,f^Within rows, different subscripts indicate significant (*P*<.05) pairwise differences between means on the Tamhane T2 post hoc test. H=high; M=medium; L=low.

A multinomial logistic regression model was used to test the association between the 3 profiles and the evolution of the number of patients treated over the past 2 years (a categorical-type variable with 3 categories, ie, 1: decrease, 2: remain the same, 3: increase) by controlling 6 characteristic variables with *stable* level as the reference for the 3-category outcome variable (Multimedia Appendix 3). This model indicates that physicians in the *weak* profile are more likely to have experienced a decrease in the number of patients treated than those in both the *strong* and *moderate* profiles, with OR 1.67 (95% CI 1.20-2.33) and OR 1.82 (95% CI 1.33-2.48), respectively.

## Discussion

Over the past several years, scholars and policy makers have agreed on the unsustainable nature of the increasing trends of health care spending and investing in health information technologies is seen as a viable option in dealing with this threat. As a result, most countries of the Organisation for Economic Co-operation and Development have begun promoting and investing in CISs and making them one of their top priorities. In this context, 4 CISs have emerged as the most important: EHR, HIE, PHR, and telehealth. Although our knowledge of the 4 CISs has been advanced by several studies that have investigated their adoption and associated performance outcomes, the majority did so by considering the 4 CISs in isolation, which implies that our understanding of this complex phenomenon is still limited.

Using data collected by the EC through a survey of 5793 GPs conducted throughout the EU, this study sought to improve our understanding of the adoption of 4 CISs and the implications for performance outcomes. To the best of our knowledge, this study is one of the first to investigate the empirical configurations of the capabilities of the 4 most important CISs (EHR, HIE, PHR, and telehealth) in general practice settings. At the same time, we believe that the findings of this study provide several interesting insights for medical informatics research by confirming, extending, or challenging previous results, in addition to having important normative implications.

First, consistent with previous studies on the adoption of EHR [67,68] or HIE [69] in practice settings or ambulatory care, our results confirm that CISs are less prevalent among older GPs, working alone in solo practices. However, we found no association with gender, and our study revealed 2 surprising results that call for further investigations: (1) GPs with more years of experience are more likely to adopt electronic data storage. Of note is the fact that the prediction of *electronic storage of patients’ data* by age and *years spent in general practice* was both weak, with ORs close to 1.0 of 0.97 and 1.02, respectively, and (2) GPs within a practice located in a medium- to small-sized city or a rural town are less likely than those in a large city to be in the *weak* profile.

Second, after measuring the capabilities associated with each of the 4 CISs, we empirically uncovered 3 theoretically meaningful and significantly well-separated configurations of profiles of CIS adoption by GPs. This result empirically confirms that CIS capabilities as organizational elements correlate in an understandable and stable way [31,70], and only a fraction of the theoretically conceivable configurations is viable and apt to be observed empirically [31].

Third, among the 3 profiles, one, the *strong* profile, outperforms the other two and leads to significantly high performance in terms of the quality of care provided to patients, efficiency of the practice, productivity of the practice, and improvement of working processes. Given that the *strong* profile scored high (H) and higher than the other 2 profiles on all 4 CIS capabilities, the contrary would have been very worrying. Similarly, when compared in terms of *improvement of working processes* as a practice performance outcome, the *moderate* cluster that scored medium on all 4 CIS capabilities outperforms the *weak* profile. Although it seems obvious to expect the *moderate* profile to outperform the *weak* profile in certain ways, given the statistically significant differences in 2 CIS capabilities (of 4), the question remains as to why the expected performance gap manifests itself in exactly one performance indicator and why this indicator is *improvement of working process* and not the *quality of care provided to patients* or the *productivity of the practice* or the *efficiency of the practice*. Again, as expected, both the *strong* and *moderate* profiles outperform the *weak* profile in the number of patients treated over the past 2 years, but surprisingly, our results revealed no significant difference between the *strong* and *moderate* profiles (Multimedia Appendix 3).

Fourth, 2 counterintuitive pictures emerged from our results. First, when scrutinizing the *weak* profile, it can be noticed that this group displays a profile that seems to be reversed in terms of CIS capabilities. In fact, it exhibits a *low* score for EHR and HIE capabilities while exhibiting a medium score for telehealth and PHR. One would expect GPs to first build strong capabilities for EHR and HIE before considering the adoption of telehealth and PHR. Second, GPs in the *weak* profile deploy an overall set of CIS capabilities that seems to be inferior to the *moderate* profile, yet achieve *equifinal* performance outcomes [31,35]. More specifically, both profiles exhibit a *medium* score on 3 performance outcomes: *productivity of the practice*, *efficiency of the practice*, and *quality of care*. Following Gruber et al [71], who obtained similar results when linking small firms’ capabilities to performance, we contend that these findings also suggest that configurations that lead to relatively higher performance outcomes for general practices in terms of the quality of care, efficiency of the practice, and productivity are not necessarily the inverse of those that lead to lower performance outcomes. It is important to remember that a general practice can be understood as a small firm.

This research contributes to the configuration literature by responding to a call for further empirical research on equifinality [35].

From a practical viewpoint, we contend that the resulting profiles of European GPs will assist policy makers to make sense of the general practice adoption of the 4 major CISs. As indicated in our study, GPs have been separated into “discrete and relatively homogeneous groups” [31] with different emphasis on EHR, HIE, PHR, and Telehealth capabilities, and unveiled associated practice performance consequences regarding five indicators. In particular, our results indicate that for a practice to maximize performance outcomes, it should develop high capabilities in all 4 CISs, because efforts to develop high capabilities on a selective subset may only be in vain, without any significant performance outcome to the point of being equivalent to developing inferior capabilities, at least concerning *quality of care provided to patients, efficiency of the practice* and *productivity of the practice*. Thus, policy makers should continue their efforts to stimulate the adoption of the 4 CISs among general practices while raising awareness of the importance of achieving a *comprehensive profile*. In addition, given the laggardness of solo practices, it could be advised to define specific initiatives targeting this category of general practice.

By investigating the configuration of the 4 most important CISs and the associated implications for performance outcomes, this study explores a topic that has received limited attention until now. Hence, in interpreting our results, one should keep in mind some limitations. First, generalizability may be limited because our sample is composed of only European GPs. Second, there are intrinsic limitations due to the use of secondary data. In fact, we used a data set that was not collected to meet the specific objectives of this study. Third, even though the results of the testing instrument reported in this study indicate high reliability for most scales, one out of the 4 scales measuring CIS capability (telehealth) has a reliability less than 0.6. In addition, even though the instrument has substantial face validity, it has not been subjected to formal psychometric assessment.

Given the paucity of studies that investigate the empirical configurations of the 4 CISs (EHR, HIE, PHR, and Telehealth) at either primary care practice or hospital settings, we encourage other researchers to build upon our results and investigate the configuration of these 4 CISs and the associated implications for performance outcomes in other regions, including hospital settings.

## Appendix

Multimedia Appendix 1 General practitioners' characteristics associated with the Adoption of electronic storage of patient data.

Multimedia Appendix 2 Results of the logistic regression of general practioners and practice characteristics by cluster.

Multimedia Appendix 3 Multinomial logistic regression model to test association between profiles and evolution of the number of patients over the past 2 years, by controlling 6 characteristic variables.

## Abbreviations

ANOVA: analysis of variance
CIS: clinical information system
DOI: diffusion of innovation
EC: European commission
eHealth: electronic health
EHR: electronic health record
EU: European Union
GP: general practitioner
HIE: health information exchange
ICT: information and communication technology
OR: odds ratio
PHR: personal health record
