# Supplementary material for: Adoption and Performance of Complementary Clinical Information Technologies: Analysis of a Survey of General Practitioners
Source: J Med Internet Res. 2020 Jul 23;22(7):e16300. doi: 10.2196/16300 (PMC7413273; doi:10.2196/16300)
Supplement: Multimedia Appendix 1 [file jmir_v22i7e16300_app1.docx]

APPENDIX 1

| GPs Characteristics Associated with The Adoption of Electronic Storage of Patient Data (n=5793) | | | | | |
| --- | --- | --- | --- | --- | --- |
| Predictor | Predictor level comparison | OR | Lower bound of 95%CI | Upper bound of 95%CI | *P*-Value |
| Gender | Female vs Male (Male as reference) | 0.851 | 0.709 | 1.023 | 0.0861 |
| Age | NA | 0.971 | 0.956 | 0.987 | 0.0003 |
| Professional status | GP working in a Health center vs Self-employed GP working alone | 0.445 | 0.289 | 0.685 | 0.0002 |
|  | Self-employed GP working in a group practice vs Self-employed GP working alone | 1.487 | 0.874 | 2.529 | 0.1433 |
|  | Other vs Self-employed GP working alone | 0.341 | 0.237 | 0.491 | <.0001 |
| Workplace location | Mid-small city vs Large city | 0.974 | 0.778 | 1.218 | 0.8148 |
|  | Rural town vs Large city | 1.109 | 0.894 | 1.376 | 0.3464 |
| Years spent if general practice | NA | 1.022 | 1.008 | 1.037 | 0.0030 |
| Practice size | 2- SMALL vs 1-SOLO_ | 2.651 | 1.669 | 4.210 | <.0001 |
|  | 3- MEDIU vs 1-SOLO_ | 3.518 | 2.209 | 5.604 | <.0001 |
|  | 4- LARGE vs 1-SOLO_ | 2.818 | 1.827 | 4.345 | <.0001 |
