# Supplementary material for: Adoption and Performance of Complementary Clinical Information Technologies: Analysis of a Survey of General Practitioners
Source: J Med Internet Res. 2020 Jul 23;22(7):e16300. doi: 10.2196/16300 (PMC7413273; doi:10.2196/16300)
Supplement: Multimedia Appendix 2 [file jmir_v22i7e16300_app2.docx]

APPENDIX 2

| Results of the Logistic Regression of GPs and Practice Characteristics by Cluster (odds ratios, OR) | | | | | | | | | | |
| --- | --- | --- | --- | --- | --- | --- | --- | --- | --- | --- |
|  |  |  | Univariate models | | | | Multivariate model | | | |
| Predictor | Predictor level comparison | Cluster I-Strong profile as reference | OR | Lower bound of 95%CI | Upper bound of 95%CI | *P*-Value | OR | Lower bound of 95%CI | Upper bound of 95%CI | *P*-Value |
| Gender | Female vs Male (Male as reference) | III | 1.528 | 1.258 | 1.857 | <.0001 | 1.520 | 1.237 | 1.868 | <.0001 |
|  |  | II | 1.086 | 0.967 | 1.219 | 0.1634 | 1.175 | 1.037 | 1.330 | 0.0114 |
| Age | NA | III | 1.001 | 0.992 | 1.010 | 0.8603 | 1.014 | 0.997 | 1.032 | 0.1070 |
|  |  | II | 0.996 | 0.991 | 1.001 | 0.1426 | 0.973 | 0.962 | 0.984 | <.0001 |
| Professional status | GP working in a Health center vs Self-employed GP working alone | III | 0.204 | 0.159 | 0.262 | <.0001 | 0.457 | 0.240 | 0.870 | 0.0171 |
|  |  | II | 0.335 | 0.289 | 0.388 | <.0001 | 0.648 | 0.423 | 0.994 | 0.0466 |
|  | Self-employed GP working in a group practice vs Self-employed GP working alone | III | 0.202 | 0.149 | 0.273 | <.0001 | 0.442 | 0.224 | 0.870 | 0.0182 |
|  |  | II | 0.556 | 0.476 | 0.650 | <.0001 | 0.845 | 0.546 | 1.308 | 0.4511 |
|  | Other vs Self-employed GP working alone | III | 0.643 | 0.460 | 0.900 | 0.0099 | 1.161 | 0.653 | 2.063 | 0.6117 |
|  |  | II | 0.627 | 0.497 | 0.791 | <.0001 | 0.977 | 0.650 | 1.468 | 0.9114 |
| Workplace location | Mid-small city vs Large city | III | 0.794 | 0.625 | 1.010 | 0.0599 | 0.741 | 0.578 | 0.950 | 0.0183 |
|  |  | II | 0.961 | 0.830 | 1.113 | 0.5997 | 0.898 | 0.771 | 1.046 | 0.1685 |
|  | Rural town vs Large city | III | 0.749 | 0.596 | 0.940 | 0.0125 | 0.604 | 0.475 | 0.768 | <.0001 |
|  |  | II | 1.117 | 0.975 | 1.279 | 0.1101 | 0.909 | 0.787 | 1.050 | 0.1948 |
| Years spent if general practice | NA | III | 0.988 | 0.979 | 0.996 | 0.0049 | 0.972 | 0.956 | 0.988 | 0.0007 |
|  |  | II | 0.997 | 0.992 | 1.002 | 0.2577 | 1.015 | 1.004 | 1.026 | 0.0080 |
| Practice size | 2- SMALL vs 1-SOLO_ | III | 0.312 | 0.228 | 0.428 | <.0001 | 0.587 | 0.303 | 1.139 | 0.1154 |
|  |  | II | 0.769 | 0.647 | 0.914 | 0.0028 | 0.907 | 0.590 | 1.394 | 0.6559 |
|  | 3- MEDIU vs 1-SOLO_ | III | 0.186 | 0.137 | 0.254 | <.0001 | 0.342 | 0.177 | 0.661 | 0.0014 |
|  |  | II | 0.382 | 0.325 | 0.450 | <.0001 | 0.462 | 0.302 | 0.707 | 0.0004 |
|  | 4-LARGE vs 1-SOLO_ | III | 0.234 | 0.180 | 0.304 | <.0001 | 0.365 | 0.194 | 0.688 | 0.0018 |
|  |  | II | 0.307 | 0.262 | 0.359 | <.0001 | 0.369 | 0.242 | 0.562 | <.0001 |
| I= Strong profile  II=Medium profile  III=Weak profile | | | | | | | | | | |
