# Supplementary material for: Adoption and Performance of Complementary Clinical Information Technologies: Analysis of a Survey of General Practitioners
Source: J Med Internet Res. 2020 Jul 23;22(7):e16300. doi: 10.2196/16300 (PMC7413273; doi:10.2196/16300)
Supplement: Multimedia Appendix 3 [file jmir_v22i7e16300_app3.docx]

APPENDIX 3

| Multinomial logistic regression model to test association between profiles and evolution of the number of patients over the past two (2) years, by controlling 6 characteristic variables. | | | | | | |
| --- | --- | --- | --- | --- | --- | --- |
| Independent variable: profile comparison | The evolution number of patients over the past two (2) years | OR | Lower bound of 95%CI | Upper bound of 95%CI | Wald Chi-Squared test | *P*-Value |
| Weak vs Strong | decrease | 1.670 | 1.199 | 2.325 | 9.2166 | 0.0024 |
| Weak vs Strong | increase | 1.119 | 0.887 | 1.412 | 0.9049 | 0.3415 |
| Medium vs Strong | decrease | 0.919 | 0.733 | 1.151 | 0.5425 | 0.4614 |
| Medium vs Strong | increase | 1.020 | 0.890 | 1.170 | 0.0825 | 0.7740 |
| Weak vs Medium | decrease | 1.817 | 1.332 | 2.480 | 14.1836 | 0.0002 |
| Weak vs Medium | increase | 1.097 | 0.879 | 1.369 | 0.6735 | 0.4118 |
